# Compensatory Failure of Autonomic Regulation in Phantom Limb Pain and Its Correlation with Maladaptive Plasticity: A Cross-Sectional HRV Study in Amputees

**DOI:** 10.3390/biomedicines13112710

**Published:** 2025-11-04

**Authors:** Nadine Aranis, Eneidy Piña Mojica, Jean Alex Matos Ribeiro, David Sparrow, David Crandell, Anna Lepesteur Gianlorenco, Felipe Fregni

**Affiliations:** 1 Neuromodulation Center and Center for Clinical Research Learning, Spaulding Rehabilitation Hospital and Massachusetts General Hospital, Harvard Medical School, Boston, MA 02138, USA; naranis@mghihp.edu (N.A.); epina7@mgb.org (E.P.M.); alepesteurgianlorenco@mgh.harvard.edu (A.L.G.); 2 Department of Physical Therapy, Federal University of São Carlos, São Paulo 13565-905, Brazil; ribeiro-matos@hotmail.com; 3 Department of Medicine, Avedisian School of Medicine and VA Normative Aging Study, Veterans Affairs Boston Healthcare System, Boston University Chobanian, Boston, MA 02130, USA; david.sparrow@va.gov; 4 Department of Physical Medicine Rehabilitation, Harvard Medical School, Spaulding Rehabilitation Hospital, Boston, MA 02115, USA; dcrandell@mgh.harvard.edu

**Keywords:** phantom limb pain, heart rate variability, autonomic nervous system, motor cortex plasticity, central autonomic network

## Abstract

**Background/Objectives**: Chronic pain is associated with autonomic nervous system (ANS) dysfunction, which can be indexed by heart rate variability (HRV). This cross-sectional study examined associations between ANS dysregulation and phantom limb pain (PLP) intensity in amputees and explored related clinical and psychosocial variables. **Methods**: Fifty-three adults with chronic PLP (mean age 57.7 ± 15.4; 60% male) were enrolled. Primary exposure was PLP intensity concurrent with HRV time- and frequency-domain metrics. Additional variables included residual limb pain, phantom limb sensations (PLS), telescoping and PLP–PLS index. **Results**: PLP intensity was not significantly associated with frequency-domain HRV measures. Time-domain parameters RMSSD (root mean square of successive differences) and pNN50 (percentage of successive normal RR intervals >50 ms) were lower in the positive PLP–PLS group. In multivariate models, depression (range of βs:−1.36 to −58.16), pain-medication use (range of βs: −12.3 to −953.4), and body mass index (range of βs: 0.55 to 45.8) were significantly associated with lower SDNN (standard deviation of NN intervals), RMSSD, pNN50, low-frequency (LF), and high-frequency (HF) power. Female sex and pre-amputation pain correlated with higher LF, whereas traumatic etiology correlated with lower LF. Poor sleep quality correlated with elevated HF. No predictors related to the LF/HF ratio. **Conclusions**: HRV alterations in PLP were associated with depression and pain-medication use rather than pain intensity. Findings support HRV dysfunction as a marker of brain compensatory failure within the autonomic circuit, and maladaptive plasticity, highlighting utility for identifying central dysfunction even when pain severity does not vary.

## 1. Introduction

Phantom limb pain (PLP) is a prevalent condition, affecting 60% to 85% of the 5.6 million people in the United States living with limb loss [1,2] and is defined as pain felt in the part of the limb that no longer remains [3]. The underlying mechanisms of PLP are multifactorial and interrelated, involving the central and peripheral nervous systems and psychosocial factors [1,2]. Current evidence suggests that central factors related to PLP arise from maladaptive cortical reorganization or plasticity within the central nervous system (CNS), particularly involving the primary somatosensory (S1) and motor (M1) cortex [2,4,5,6,7], or reduced interregional functional connectivity despite preserved brain structure organization [6,7]. Moreover, disruption of peripheral afferent input leads to abnormal excitability and reorganization within the M1 and other brain regions [6,8,9]. The involvement of asymmetrical motor areas in pain perception supports the hypothesis that PLP may be at least partly modulated by motor cortical function, highlighting a potential role for the autonomic nervous system (ANS) in this process [4], and supporting the need to explore other neurophysiological contributors to pain, beyond cortical reorganization that may contribute to the onset and intensity of pain [10].

The ANS plays a central role in pain modulation and has been implicated in the pathophysiology of multiple chronic pain conditions [11,12]. Dysregulation within the central autonomic network (CAN)—which comprises interconnected cortical, subcortical, and brainstem structures—can enhance pain perception and impair descending inhibitory mechanisms [12,13]. In the context of PLP, mounting evidence suggests that CAN dysfunction may not simply coexist with pain but instead arise from shared neurobiological alterations, such as cortical disinhibition, deafferentation, and aberrant thalamocortical signaling. These maladaptive changes, which follow amputation, may simultaneously disrupt autonomic regulation and amplify nociceptive processing. Supporting this notion, studies in amputees have documented elevated norepinephrine levels and increased α-1 adrenergic receptor expression—findings consistent with sympathetic overactivity that could both result from and reinforce central maladaptive plasticity [14,15]. These data raise the possibility that autonomic dysfunction and PLP may reflect parallel manifestations of a common underlying brain-based compensatory failure. Indeed, some studies in chronic pain did not find a correlation between pain severity and HRV measures [16].

Heart rate variability (HRV) provides a non-invasive biomarker of autonomic function by measuring the oscillations in R-R intervals (RRi) over time. Lower HRV is typically interpreted as a marker of reduced parasympathetic (vagal) activity and/or elevated sympathetic tone [17]. In recent years, HRV has been used to assess the autonomic correlates of chronic pain syndromes, including migraine [18], fibromyalgia [19,20], and posttraumatic stress disorder [21]. HRV has also shown utility in evaluating treatment efficacy and predicting pain trajectories [22,23].

Despite these promising insights, there remains a paucity of studies examining HRV in individuals with chronic PLP. To our knowledge, there is only one study on male amputees that reports decreased HRV, regardless of whether PLP is present or not [24]. Given the potential of HRV to reflect alterations in autonomic regulation, further investigation in this population is warranted. This study aims to evaluate ANS activity—measured via HRV—in individuals with chronic PLP following amputation. We hypothesize that reduced HRV parameters will be associated with worse clinical indicators, indicating a dysregulated autonomic profile in this population.

## 2. Materials and Methods

### 2.1. Study Design and Participants

This is a cross-sectional study analysis of amputee patients affected by chronic PLP (*n* = 53) from an ongoing parallel randomized clinical trial study, “Pragmatic Trial of Remote tDCS and Somatosensory Training for Phantom Limb Pain with Machine Learning to Predict Treatment Response (PLP-EVEREST trial)”. This NIH-funded clinical trial (Grant 2R01-HD082302-06) is being conducted in the Neuromodulation Center, Spaulding Rehabilitation Hospital (Boston, MA, USA), following the local research ethics regulations and the Declaration of Helsinki.

Patients from across the United States were eligible for the study if they were 18 years of age or older, had an acquired amputation of one or more limbs, had current chronic pain with an average pain intensity of at least four on the numeric rating scale (NRS, 0: no pain, 10: worst imaginable pain), and experienced PLP regularly (at least once a week). Participants were required to sign the informed consent form to be included in the study. Subjects were excluded if they had any clinically significant, unstable medical or psychiatric disorder, history of substance abuse in the past 6 months (according to the DSM-V criteria for substance use disorder with six or more symptoms), uncompensated psychiatric disorder, previous significant neurological history with current significant neurological deficits, or previous neurosurgical procedure with craniectomy.

### 2.2. Main Outcomes

ANS response based on HRV linear analysis represented the cardiac parasympathetic and sympathetic activity.

RRi were recorded using a Polar H10 band positioned on the patient’s chest, at the lower third of the sternum. A trained physician oversaw the procedure, ensuring participants remained at rest for 5 min and breathed at their normal rate. Participants were advised to avoid caffeine and smoking at least two hours before testing. When feasible, recordings were scheduled in the morning hours to minimize circadian effects. The data were then transferred to Microsoft Excel for preprocessing. The RRi average was computed by calculating the mean of the central segment of normal RRi. Any intervals that fell below 80% or above 120% of the RRi average were identified as ectopic beats. These were removed from the series and replaced with the mean of the preceding and subsequent acceptable intervals, following the guidelines of the European Society of Cardiology and the North American Society of Pacing and Electrophysiology [17]. RRi series with a variation exceeding 20% were excluded from further analysis. Finally, the data were processed using Kubios HRV Scientific Software (version 2.2, Matlab, Kuopio, Finland), with only the most stable sections, containing 256 points, selected for HRV analysis in both time and frequency domains.

Time-domain indices included: (1) standard deviation of normal-to-normal RRi (SDNN), (2) root mean square of successive RRi differences (RMSSD), and (3) percentage of successive normal RR intervals more than 50 ms (pNN50). The SDNN reflects all the cyclic components responsible for variability in the period of recording, while indices of cardiac parasympathetic tone included in this domain are RMSSD and pNN50. The frequency-domain indices were derived using the Fast Fourier Transformation (FFT) to compute the power frequency spectrum from 0.00 to 0.40 Hz, separately for the low frequency (LF) in the range 0.04–0.15 Hz and the high frequency (HF) in the range 0.15–0.40 Hz, as absolute values of variance in milliseconds squared (ms^2^). The LF reflects the sympathetic modulation of the ANS, while the HF reflects the parasympathetic tone of the ANS. The LF/HF ratio will also be considered to represent the controlled and balanced behavior of the two branches [25].

### 2.3. Main Exposure

We examine the distribution of pain intensity in our population of chronic PLP patients measured by the visual analog scale (VAS) at the same moment that HRV metrics were obtained. This tool is validated for use in chronic pain, and scores are recorded on a 10 cm line representing a continuum between “no pain” and “worst pain.” Since multiple post-amputation pain syndromes require differentiation from PLP, residual limb pain, phantom limb sensations (PLS), and telescopic pain were also assessed by VAS and considered for further exploration during the analysis. To characterize clinical sub-phenotypes within amputee patients, Ortega-Márquez et al. reported the PLP-PLS index (PLP intensity minus PLS intensity), which can be used to estimate the level of the sensorimotor maladaptive process (positive values suggesting maladaptation, wherein pain prevails over phantom sensation) [26].

### 2.4. Covariates

Sociodemographic covariates include age, sex, body mass index (BMI), race, ethnicity, and educational level (high school, undergraduate or graduate degree). Medical history covariates include pain medication, PLP frequency, time since amputation, amputation side (left or right), site (lower or upper extremity) and level, reason for amputation (i.e., traumatic, infection, diabetes, or other), and use of prosthesis. Psychosocial covariates include pain catastrophizing, anxiety, depression, cognition, and sleep quality. Further details on the conceptual and operational definitions of these variables are summarized in Table S1.

### 2.5. Statistical Analyses

All available observations are being included for descriptive purposes, following a complete-case analysis approach. The proportion of missing data is minimal (primarily missing covariates) and unlikely to impact the findings. Data are summarized with mean and standard deviation (SD) for normally distributed variables and median and interquartile range (IQR) for non-parametric variables. Categorical variables are described in terms of frequency and proportions.

We conducted a univariate linear analysis to identify potential covariates for inclusion in our multivariate analysis (selecting variables with *p* < 0.25 initially). The purposeful selection approach for model building was applied [27], selecting variables with *p* < 0.05 from sociodemographic, medical history, pain-related assessments, and psychosocial variables. Statistically significant variables improved the adjusted R2, or those identified as potential confounders were added to the multivariate model. Potential confounders are defined as variables that alter the β coefficient for the exposure variable by more than 10% when added to a particular model. The assumptions of linearity and homoscedasticity were tested through graphical assessment. All analyses were conducted with R Statistical Software (v4.1.2; R Core Team 2024).

## 3. Results

### 3.1. Sample Characteristics and PLP-PLS Index—Group Comparisons

Participants (*n* = 53) had a mean age of 57.72 years (SD = 15.40) and were predominantly male (60%). Their BMI was 29.26 kg/m^2^ (SD = 6.76), and the median time since amputation was 3.5 years (IQR 2–9 years). Regarding pain characteristics, most participants reported experiencing PLP several times per day (55%), and 77% were taking pain medications. Among medication users (*n* = 41), some participants reported more than one medication type. More than 60% were taking gabapentin (*n* = 35), followed by analgesics (*n* = 18), opioids (*n* = 9), and antidepressants (*n* = 1). Additionally, 74% of participants had used or were currently using a prosthesis at the time of assessment. No statistically significant differences were observed between the positive (*n* = 29) and negative (*n* = 24) PLP-PLS index groups. Further sociodemographic, medical history, and pain-related characteristics are described in Table 1. The distribution of psychosocial variables is presented in Figure S1.

### 3.2. Univariate Analyses

Univariate linear regressions were conducted to examine the association of each predictor with the HRV outcomes (Tables S2 and S3). Age was positively associated with Mean RRi (β = 2.74, *p*-value = 0.02) and LF (β = 0.23, *p*-value = 0.01). BMI was inversely related to HF power (β = −0.25, *p*-value = 0.005) and positively related to the LF/HF ratio (β = 0.22, *p*-value = 0.009), and participants with a undergraduate degree had reduced pNN50 (β = −7.66, *p*-value = 0.03) and lower LF power (β = −0.30, *p*-value = 0.002), compared to high school educated individuals.

Regarding the amputation site, upper limb amputations predicted a higher LF/HF ratio (β = 3.86, *p*-value = 0.09). Sepsis-related amputation was associated with reduced HF power (β = −0.20, *p*-value = 0.015), and traumatic etiology was associated with decreased SDNN (β = −6.92, *p*-value = 0.007) and RMSSD (β = −1.16, *p*-value = 0.01), but increased LF (β = 0.21, *p*-value = 0.009). The use of pain medication was related to lower pNN50 (β = −9.15, *p*-value = 0.01), and to lower HF power (β = −0.22, *p*-value = 0.007).

Among psychosocial variables, higher anxiety scores corresponded to greater RMSSD (β = 0.17, *p*-value = 0.04), and HF (β = 0.17, *p*-value = 0.03), and depression score was inversely related to the LF/HF ratio (β = −0.15, *p*-value = 0.03). No other predictors reached statistical significance (*p*-value > 0.05), but following the purposeful selection approach for model building, we considered predictors with a *p*-value < 0.25 for inclusion in the multivariate analyses.

### 3.3. Multivariate Analyses

The multivariate linear regression models predicting HRV time-domain measures are presented in Table 2. The model for Mean RRi was not significant (R^2^ = 0.04). In contrast, the SDNN model accounted for 17% of the variance, and showed that higher depression score was independently associated with lower SDNN (β = −1.36, 95%CI [−2.71, −0.01], *p*-value = 0.04) as was use of pain medication (β = −20.49, 95%CI [−35.59, −5.39], *p*-value = 0.009). The RMSSD model explained 30% of the variance, with BMI (β = 1.46, 95%CI [0.34, 2.57], *p* = 0.01), depression score (β = −1.55, 95%CI [−2.93, −0.18], *p*-value = 0.02), and pain medication use (β = −25.86, 95%CI [−41.04, −10.69], *p*-value = 0.001) emerging as significant predictors. Finally, the pNN50 model accounted for 23% of variance, with BMI (β = 0.55, 95%CI [0.03, 1.07], *p*-value = 0.04) and pain medication use (β = −12.30, 95%CI [−19.25, −5.36], *p*-value < 0.001) independently predicting pNN50. PLP intensity was not significantly related to any of the HRV time-domain measures.

Frequency-domain multivariate models for HRV outcomes are presented in Table 3. The LF model explained 34% of the variance, identifying female sex (β = 641.97, 95%CI [119.53, 1164.40], *p*-value = 0.01), BMI (β = 45.83, 95%CI [2.88, 88.77], *p*-value = 0.03), use of pain medication (β = −953.43, 95%CI [−1523.86, −382.99], *p* = 0.002), traumatic etiology (β = −820.52, 95%CI [−1383.37, −257.66], *p*-value = 0.005), pain before amputation (β = 752.10, 95%CI [172.18, 1332.01], *p*-value = 0.01), and depression (β = −58.16, 95%CI [−112.07, −4.25], *p*-value = 0.03) as independent predictors. The HF model accounted for 35% of variance, with BMI (β = 35.46, 95%CI [13.27, 57.64], *p*-value = 0.002), use of pain medication (β = −473.01, 95%CI [−767.56, −178.45], *p*-value = 0.002), depression (β = −38.68, 95%CI [−65.65, −11.7], *p*-value = 0.006), and sleep quality (β = 33.4, 95%CI [1.58, 65.22], *p*-value = 0.04) emerging as significant. The LF/HF ratio model explained only 10% of variance, and no predictors reached significance (all *p* > 0.05). In addition, PLP intensity was not significantly related to any of the HRV frequency-domain measures.

### 3.4. PLP-PLS Index and HRV

**Figure 1:**
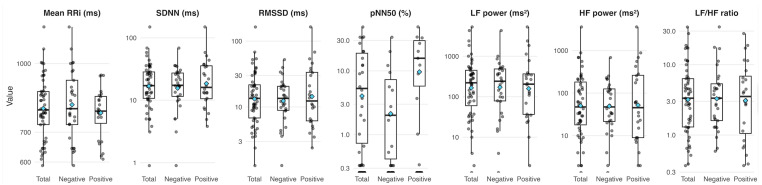
Distribution of heart rate variability (HRV) time- and frequency-domain measures across PLP–PLS index groups.

Descriptive comparisons of HRV indices between individuals with a positive versus negative PLP-PLS index revealed no statistically significant group differences across time or frequency-domain metrics (Figure 1). The median of the Mean RRi was comparable between the positive (781.00 ms [712.00–844.50]) and the negative PLP-PLS index group (791.00 ms [728.00–917.00]), *p*-value = 0.40). Similarly, no significant differences were observed between positive versus negative PLP-PLS index for SDNN (16.05 ms [10.40–36.15] vs. [10.40–36.15] vs. 17.30 ms [11.50–27.30], *p*-value = 0.70), RMSSD (12.40 ms [6.10–34.05] vs. [6.10–34.05] vs. 13.50 ms [8.90–20.60], *p*-value = 0.80), or pNN50 (0.00 [0.00–14.05] vs. [0.00–14.05] vs. 0.33 [0.00–2.01], *p*-value = 0.60). Although, a possible trend towards a lower value of RMSSD and pNN50 are found in the patients with positive PLP-PLS index, compared to the negative PLP-PLS index. Frequency-domain measures also showed no significant differences: LF power (204.50 ms^2^ [36.00–374.00] vs. 229.00 ms^2^ [77.00–478.00], *p*-value > 0.90), HF power (47.00 ms^2^ [9.00–268.50] vs. 43.00 ms^2^ [20.00–122.00], *p*-value = 0.80), and the LF/HF ratio (3.55 [0.99–7.33] vs. [0.99–7.33] vs. 3.33 [1.60–5.34], *p*-value > 0.90) did not differ meaningfully between groups. However, it is important to highlight that HF power was slightly higher in the PLP-PLS positive index group (47.00 ms^2^) compared to the negative group (43.00 ms^2^).

### 3.5. PLP-PLS Index and Psychosocial Covariates

No significant differences were found between PLP-PLS index groups across psychosocial scales (all *p*-values > 0.05; Table 1). Depression scores averaged 8.14 (SD = 4.95) in the negative PLP-PLS index group and were marginally higher, 8.92 (SD = 6.51) in the positive PLP-PLS group. Mean scores on anxiety, cognition, and sleep quality were similar between groups. However, pain catastrophizing averaged 14.64 (SD = 12.67) in the negative PLP-PLS index group and was lower in the positive PLP-PLS index group with a mean of 11.29 (SD = 10.65).

## 4. Discussion

The central premise of this study is that individuals with PLP who exhibit dysfunctional HRV are likely experiencing maladaptive brain reorganization. Despite the fact our multivariate models accounted for a modest proportion of variance (R^2^ = 0.10–0.35), findings support this hypothesis. The multifactorial nature of HRV regulation, influenced by physiological, behavioral, and environmental factors revealed significant associations between HRV indices—such as RMSSD, SDNN, and HF—and several clinical variables including BMI, amputation etiology, depression, sleep quality, and the use of pain medications. Notably, reduced HRV was observed in participants with higher depression scores and among those using pain medications, patterns consistent with dysfunction of the CAN. Together, these results suggest that HRV can serve as a physiological marker of maladaptive neuroplastic changes in the brain following limb loss.

Heart rate variability, particularly reduced parasympathetic indices such as RMSSD and HF, is increasingly recognized as a biomarker of reduced top-down control from cortical structures, especially the medial prefrontal cortex (mPFC) and anterior cingulate cortex (ACC) [28,29]. This relationship forms the foundation of the neurovisceral integration model, which posits that the same neural networks responsible for emotional and cognitive regulation also govern autonomic control [30,31]. This aligns with our data showing reduced HRV across multiple metrics (especially RMSSD, SDNN, HF) being consistently associated with higher depression scores and use of pain medication, aligning with dysfunction in the CAN.

We propose that impaired HRV reflects reduced integrative regulation between cortical pain processing and autonomic control centers, mirroring the maladaptive reorganization observed in PLP, and specifically the PLP-PLS index, when this has a positive value. This “autonomic inflexibility” may represent both a risk factor for and a consequence of chronic pain development, creating a self-reinforcing cycle of dysregulation [32,33].

Our results differ from prior evidence linking elevated BMI only with sympathetic predominance [34]. Although we observed an association between elevated BMI and LF power—an indicator of sympathetic activity, we also observed such an association with RMSSD, pNN50, and HF—key indicators of parasympathetic activity. The extensive literature suggests that higher BMI disrupts pain-inhibitory processes and is associated with worse clinical outcomes in chronic pain conditions. For instance, recent studies from a cohort have shown that elevated BMI attenuates the beneficial effects of conditioned pain modulation (CPM) on depression, physical functioning, and symptom severity in patients with knee osteoarthritis and fibromyalgia [35,36,37]. Furthermore, Imamura et al., 2025 [38], demonstrated a nonlinear, inverted U-shaped association between BMI and pain pressure threshold (PPT), with optimal PPT values peaking at a BMI of approximately 27 kg/m^2^. While we anticipated a similar disruptive effect of BMI on autonomic function, our sample had a mean BMI approaching the overweight threshold, but still within the non-obese range. This nuance may explain the positive association with HRV and suggests that the BMI-HRV relationship may follow a nonlinear trajectory, where deviations in either direction from normal BMI impair autonomic and pain-regulatory systems. Nevertheless, these results support the growing recognition that moderate BMI may confer physiological resilience in some domains, including autonomic regulation. In addition, this duality aligns with findings from Amekran et al. (2024) and Adlan et al. (2017), who noted that HRV changes in obesity often show mixed patterns depending on which indices are used and whether absolute or normalized values are reported [25,39]. Further exploration of nonlinear HRV dynamics may help clarify these divergent trends.

Based on the prior literature, we expected to find a negative association between chronic pain intensity and HRV, where greater pain would correlate with reduced parasympathetic activity and heightened sympathetic regulation. Indeed, studies such as those conducted by Koenig et al. (2016) and Tracy et al. (2016) have shown that reduced HRV is linked to increased pain sensitivity and impaired descending pain modulation in chronic pain populations [23,40]. However, in our study, we did not observe a significant association between HRV measures and PLP intensity. This absence of correlation may reflect that all participants in our study sample experienced PLP, thereby limiting the variance in PLP intensity and potentially masking any dose–response relationship between HRV and symptom severity. In this context, HRV dysfunction may be more reflective of the presence or absence of PLP rather than its intensity, capturing a broader autonomic shift associated with the neuropathic pain phenotype. This aligns with the idea that PLP arises from complex central and peripheral maladaptive processes—including cortical reorganization, thalamocortical dysrhythmia, and sympathetic hyperexcitability [5,7,13]—which may not track linearly with pain intensity, and which are more effectively indexed by dichotomous or threshold-level changes in autonomic function. Conversely, the trend towards lower RMSSD and pNN50 in patients with a positive PLP-PLS index suggests that this indicator could potentially represent a more accurate and sensitive indicator of variations in symptom severity that correlate with maladaptive reorganization.

Interestingly, our study replicated the findings from Cachadiña et al. (2013), who reported diminished HRV in male amputees irrespective of PLP status [24]. This supports the notion that amputation itself, rather than PLP intensity alone, may lead to chronic ANS alterations. Moreover, we found that the use of pain medication, was associated with reduced HRV measured by time-domain metrics (lower SDNN, RMSSD, and pNN50) and increased sympathetic dominance (higher LF power), consistent with prior studies [41,42], which showed that analgesics can modulate ANS balance, due to diminished vagal tone, either by the suppressive effect of analgesics on ANS activity or reverse causality, where those with lower HRV and more ANS dysfunction require greater pharmacological support. Most participants in our study were taking gabapentin, which has mixed evidence regarding its autonomic effects. While therapeutic doses have been shown to improve HRV parameters, particularly SDNN and HF, by attenuating sympathetic hyperexcitability [43], prolonged use may also blunt overall autonomic reactivity through central inhibitory mechanisms.

Importantly, our multivariate models identified that depression was inversely related to time- and frequency-domain HRV measures, particularly SDNN, RMSSD, and HF power, vagally mediated, and LF power, mostly associated with a sympathetic predominance. These blunted results could indicate that chronic pain and mood disorders co-occur with ANS hypo-responsivity, or that psychological comorbidities may be a stronger predictor of ANS dysregulation than PLP itself. Tracy et al. (2016) [40] and Zeid et al. (2024) [44] noted that mood disorders such as depression contribute to autonomic dysfunction, marked by lower HRV and impaired cardiovascular adaptability. This may reflect shared neurobiological pathways involving the prefrontal cortex and limbic structures that regulate both mood and autonomic outflow. Their shared limbic–autonomic circuits, including the anterior cingulate and prefrontal cortex, are areas also implicated in descending pain control [45,46,47].

### Strengths and Limitations

The cross-sectional design of this study limits causal inference and precludes consideration of the directionality of associations between any of the predictors and HRV. Additionally, HRV serves as an indirect physiological marker of central autonomic activity and should not be interpreted as direct evidence of central nervous system circuit dysregulation or compensatory failure. Although standardized instructions were provided, we recognize some inconsistency throughout the HRV recordings concerning positions, acclimatization, and time of assessment, which might influence cardiovascular reactivity. The influence of these potential confounders could not be fully eliminated and may introduce partial variability in HF and LF estimates. Moreover, there is still a lack of consensus on how HRV data should be acquired, processed, analyzed, and reported to allow the best results and posterior comparison within and between subjects [46]. Therefore, we used a standardized data processing and analysis approach after recording, involving an estimate of respiratory frequency derived from the RRi using proprietary software (Kubios). Further research is needed to investigate the potential of HRV-based predictive models’ implications of reduced HRV in pain, and interventional studies using strategies to potentiate the excitatory pathways are required in PLP patients to confirm whether greater pain intensity contributes to the autonomic dysfunction reported in such patients.

## Figures and Tables

**Table 1:** Participants’ demographics and clinical characteristics.

Variables	Total (*n* = 53)	Negative PLP-PLS Index (*n* = 24)	Positive PLP-PLS Index (*n* = 29)	*p*-Value
**Age (yrs)**	57.72 ± 15.40	59.24 ± 13.39	55.88 ± 17.65	0.4
**Sex, male**	32 (60%)	17 (59%)	15 (63%)	>0.9
**BMI (kg/m^2^)**	29.26 ± 6.76	28.89 ± 6.87	29.74 ± 6.74	0.7
**Race**				0.6
White	38 (72%)	20 (69%)	18 (75%)	
African American	7 (13%)	5 (17%)	2 (8.3%)	
More than 1 race	6 (11%)	3 (10%)	3 (13%)	
Asian	1 (1.9%)	0 (0%)	1 (4.2%)	
Not reported	1 (1.9%)	1 (3.4%)	0 (0%)	
**Ethnicity**				>0.9
Not Hispanic or Latino	46 (87%)	25 (86%)	21 (88%)	
Hispanic or Latino	7 (13%)	4 (14%)	3 (13%)	
**Educational level**				0.2
High School	14 (26%)	5 (17%)	9 (38%)	
Undergraduate	35 (66%)	21 (72%)	14 (58%)	
Graduate Degree	4 (7.5%)	2 (10%)	1 (4.2%)	
**Pain medication, yes**	41 (77%)	24 (83%)	17 (71%)	0.5
**Time since amputation (yrs)**	3.50 (2–9)	5.00 (2–12.50)	3.00 (2–5.50)	0.11
**Amputation side**				0.8
Right	24 (45%)	14 (48%)	10 (42%)	
Left	29 (55%)	15 (52%)	14 (58%)	
**Amputation site**				>0.9
Lower limb	41 (77%)	22 (76%)	19 (79%)	
Upper limb	12 (23%)	7 (24%)	5 (21%)	
**Amputation level**				0.5
Digits	6 (11%)	4 (14%)	2 (8.3%)	
Partial foot	1 (1.9%)	1 (3.4%)	0 (0%)	
Below knee	19 (36%)	9 (31%)	10 (42%)	
Knee disarticulation	2 (3.8%)	1 (3.4%)	1 (4.2%)	
Above knee	16 (30%)	11 (38%)	5 (21%)	
Hip disarticulation	3 (5.7%)	1 (3.4%)	2 (8.3%)	
Hemipelvectomy	1 (1.9%)	0 (0%)	1 (4.2%)	
Upper arm	2 (3.8%)	0 (0%)	2 (8.3%)	
Lower arm	1 (1.9%)	1 (3.4%)	0 (0%)	
Other	2 (3.8%)	1 (3.4%)	1 (4.2%)	
**Traumatic amputation, yes**	31 (58%)	15 (52%)	16 (67%)	0.4
**Reason for amputation**				>0.9
Traumatic	21 (40%)	13 (45%)	8 (33%)	
Infection	17 (32%)	9 (31%)	8 (33%)	
Diabetes	7 (13%)	3 (10%)	4 (17%)	
Cancer	2 (3.8%)	1 (3.4%)	1 (4.2%)	
Vascular	2 (3.8%)	1 (3.4%)	1 (4.2%)	
Other	4 (7.5%)	2 (6.9%)	2 (8.3%)	
**Pain before amputation, yes**	29 (55%)	16 (55%)	13 (54%)	>0.9
**PLP frequency**				0.9
A few times a year	1 (1.9%)	0 (0%)	1 (4.2%)	
A few times a month	3 (5.7%)	2 (6.9%)	1 (4.2%)	
A few times a week	11 (21%)	6 (21%)	5 (21%)	
A few times a day	29 (55%)	16 (55%)	13 (54%)	
A few times per hour	3 (5.7%)	2 (6.9%)	1 (4.2%)	
Always	6 (11%)	3 (10%)	3 (13%)	
**Residual limb pain intensity**	3.19 ± 3.10	3.26 ± 3.43	3.13 ± 2.73	0.9
**Telescopic pain**	5.00 (5.00, 5.20)	5.00 (5.00, 5.20)	5.00 (5.00, 6.70)	0.5
**Prosthesis use, yes**	39 (74%)	21 (72%)	18 (75%)	>0.9

Data are presented in mean ± SD, median (IQR), or absolute and relative (%) frequencies—Wilcoxon rank sum test, paired-samples *t*-test, or Chi-squared test performed for differences within groups.

**Table 2:** Multivariate linear models of the association between HRV time-domain outcomes and phantom limb pain-related characteristics.

Variable	Mean RR Interval (*R*^2^ = 0.04)		SDNN (*R*^2^ = 0.17)		RMSSD (*R*^2^ = 0.30)		pNN50 (*R*^2^ = 0.23)	
	β Coefficient (95%CI)	*p*-Value	β Coefficient (95%CI)	*p*-Value	β Coefficient (95%CI)	*p*-Value	β Coefficient (95%CI)	*p*-Value
Phantom limb pain intensity	1.46 (−0.15, 3.07)	0.07	−0.06 (−0.33, 0.2)	0.62	−0.07 (−0.33, 0.2)	0.61	−0.01 (−0.14, 0.11)	0.823
Age	2.2 (−0.57, 4.96)	0.11	−0.2 (−0.67, 0.26)	0.38				
BMI	−3.65 (−10.48, 3.18)	0.28	1.01 (−0.12, 2.13)	0.07	1.46 (0.34, 2.57)	0.01 *	0.55 (0.03, 1.07)	0.038 *
Depression	1.95 (−6.14, 10.04)	0.62	−1.36 (−2.71, −0.01)	0.04 *	−1.55 (−2.93, −0.18)	0.02 *	−0.56 (−1.19, 0.07)	0.082
Anxiety	−2.2 (−8.18, 3.78)	0.46	0.1 (−0.93, 1.14)	0.83	0.36 (−0.67, 1.39)	0.48	0.22 (−0.24, 0.68)	0.34
Pain catastrophizing	1.62 (−2.2, 5.43)	0.39	0.22 (−0.44, 0.87)	0.50	0.25 (−0.43, 0.92)	0.46	0.03 (−0.26, 0.33)	0.812
Cognition	−3.79 (−23.4, 15.81)	0.69	−0.94 (−4.18, 2.3)	0.56	−1.68 (−4.59, 1.22)	0.24	−0.71 (−2.06, 0.65)	0.3
Sleep quality	−8.09 (−17.73, 1.55)	0.09	1.21 (−0.4, 2.82)	0.13	1.33 (−0.28, 2.93)	0.10	0.54 (−0.21, 1.29)	0.15
Use of pain medication			−20.49 (−35.59, −5.39)	0.009 *	−25.86 (−41.04, −10.69)	0.001 *	−12.3 (−19.25, −5.36)	<0.001
Use of prosthesis			13.95 (−1.18, 29.08)	0.07	9.84 (−6.59, 26.27)	0.23		
Left amputation					7.05 (−7.06, 21.16)	0.31	4.7 (−1.31, 10.72)	0.122

CI: Confidence Interval. * *p* < 0.05.

**Table 3:** Multivariate linear models of the association between HRV frequency-domain outcomes and phantom limb pain-related characteristics.

Variable	Low Frequency (*R*^2^ = 0.34)		High Frequency (*R*^2^ = 0.35)		LF/HF Ratio (*R*^2^ = 0.10)	
	β Coefficient (95%CI)	*p*-Value	β Coefficient (95%CI)	*p*-Value	β Coefficient (95%CI)	*p*-Value
Phantom limb pain intensity	−0.71 (−11.02, 9.6)	0.89	−1.94 (−7.29, 3.4)	0.467	0.03 (−0.05, 0.11)	0.40
Female	641.97 (119.53, 1164.4)	0.01 *	213.9 (−54.77, 482.57)	0.12		
BMI	45.83 (2.88, 88.77)	0.03 *	35.46 (13.27, 57.64)	0.002 *		
Use of pain medication	−953.43 (−1523.86, −382.99)	0.002 *	−473.01 (−767.56, −178.45)	0.002 *		
Traumatic amputation	−820.52 (−1383.37, −257.66)	0.005 *				
Pain before amputation	752.1 (172.18, 1332.01)	0.01 *				
Use of prosthesis	530.31 (−64.88, 1125.5)	0.07	282.74 (−22.96, 588.45)	0.06		
Depression	−58.16 (−112.07, −4.25)	0.03 *	−38.68 (−65.65, −11.7)	0.006 *	0.19 (−0.2, 0.58)	0.33
Anxiety	−25.27 (−70.03, 19.49)	0.26	−3.51 (−26.84, 19.82)	0.70	−0.26 (−0.54, 0.02)	0.06
Pain catastrophizing	10.67 (−15.8, 37.15)	0.41	6.28 (−6.81, 19.37)	0.33		
Cognition	−14.03 (−127.59, 99.53)	0.80	−34.45 (−93.35, 24.45)	0.24	−0.03 (−0.96, 0.9)	0.95
Sleep quality	44.93 (−17.52, 107.39)	0.15	33.4 (1.58, 65.22)	0.04 *	0.42 (−0.03, 0.87)	0.06
Age			−8.18 (−18.57, 2.21)	0.12	−0.08 (−0.22, 0.06)	0.23

CI: Confidence Interval. * *p* < 0.05.

## Data Availability

Data available within the article or its Supplementary Materials.

## Supplementary Materials

The following supporting information can be downloaded at https://www.mdpi.com/article/10.3390/biomedicines13112710/s1, Table S1. Conceptual, operational, and statistical definitions of the variables; Figure S1. Distribution of psychosocial variables across PLP–PLS index groups; Table S2. Univariate analyses for HRV time domain outcomes; Table S3. Univariate analyses for HRV frequency domain outcomes.

## Abbreviations

The following abbreviations are used in this manuscript:

CAN	Central Autonomic Network
PLP	Phantom Limb Pain
PLP-PLS Index	Phantom Limb Pain-Phantom Limb Sensation Index
HF	High Frequency
HRV	Heart Rate Variability
LF	Low Frequency
pNN50	Percentage of Successive Normal RR intervals >50 ms
RMSSD	Root Mean Square of Successive Differences
RRi	R-R interval
SDDN	Standard Deviation of NN Intervals
