# Supplementary material for: Compensatory Failure of Autonomic Regulation in Phantom Limb Pain and Its Correlation with Maladaptive Plasticity: A Cross-Sectional HRV Study in Amputees
Source: Biomedicines. 2025 Nov 4;13(11):2710. doi: 10.3390/biomedicines13112710 (PMC12650142; doi:10.3390/biomedicines13112710)
Supplement: Supplementary file 1 [file biomedicines-13-02710-s001.zip › biomedicines-3892039-supplementary.pdf]

Table S1. Conceptual, operational, and statistical definitions of the variables.

Dimension	Variable	Conceptual definition	Operational definition	Statistical definition
**Socio-demographics**	Age	Year of age at the time of the assessment.	Measured in years	Continuous
	Sex	A person's biological characteristics, self-reported.	0 = male; 1 = female	Binary, categorical
	BMI	Calculation that estimates body fat based on a person's height and weight.	Numerical index	Continuous
	Race	Person's self-identification of race, self-reported.	0 = white; 1 = black or african american, 2 = more than one race, 3 = asian, 4 = unknown or not reported	Nominal, categorical
	Ethnicity	Person's cultural identity, self-reported.	0 = not hispanic or latino; 1 = hispanic or latino; 2 = unknown or not reported	Nominal, categorical
	Educational level	Amount of formal education a person has received, self-reported.	0 = high school; 1 = Undergraduate; 2 = Graduate degree	Ordinal, categorical
**Medical history**	Pain medication	Pain medication currently used.	0 = no; 1 = yes	Binary, categorical
	Time since amputation	Years elapsed since last amputation date.	Measured in years.	Continuous
	Amputation side	Anatomical location along the limb where the residual limb terminates after surgical removal of tissue or bone, considering the last amputation.	0 = right; 1 = left	Binary, categorical
	Amputation site		0 = lower limb; 1 = upper limb	Binary, categorical
	Amputation level		1 = digits; 2 = partial foot; 3 = ankle disarticulation; 4 = below the knee; 5 = knee disarticulation; 6 = above the knee (transfemoral); 7 = hip disarticulation; 8 = hemipelvectomy/ hindquarter amputation; 9 = upper arm/shoulder; 10 = lower arm; 11 = other	Nominal, categorical
	Reason for amputation	The primary etiological condition or event that necessitated limb removal.	0 = traumatic; 1 = sepsis/infection/diabetes; 2 = cancer; 3 = vascular anomaly, non-diabetic; 4 = aortic shooting/vascular/arterial damage/circulation problem; 5 = other	Nominal, categorical
	Prosthesis use	Whether the individual currently uses or has relied in the past on a prosthetic limb for any purposeful activity in daily life.	0 = no; 1 = yes	Binary, categorical
**Pain related-variables**	Phantom limb pain frequency	Frequence that patients experience phantom pain.	1 = never; 2 = a few times a year; 3 = a few times a month; 4 = a few times a week; 5 = a few times a day; 6 = a few times per hour; 7 = always	Nominal, categorical

	Phantom limb pain	Painful sensation referring to the missing limb.	0 to 10 scale, 0 - indicating no pain at all and 10 - the worst pain felt.	Continuous
	Residual limb pain	Pain in the area adjacent to the amputated body part, or in the stump.	0 to 10 scale, 0 - indicating no pain at all and 10 - the worst pain felt.	Continuous
	Phantom sensation	Non-painful sensations referring to the missing limb	0 to 10 scale, 0 - indicating no phantom limb sensation and 10 - full sensation of the amputated limb.	Continuous
	Telescopic pain	Shrinking and retraction of the phantom towards the residual limb.	0 to 10 scale, 0 - indicating the phantom was enlarged and 10 - the phantom was completely retracted into the stump.	Continuous
**HRV outcomes**	Mean R-R interval	Mean of all normal-to-normal (NN) intervals obtained from a 5-min resting ECG, following artifact exclusion per the Task Force HRV guidelines.	Measured in milliseconds (ms)	Continuous
	Standard Deviation of NN intervals (SDNN)	Variability in the time intervals between consecutive heartbeats.	Measured in milliseconds (ms)	Continuous
	Root Mean Square of Successive Differences (RMSSD)	Reflects the short-term variability in HR, focusing on the beat-to-beat differences in consecutive NN (or RR) intervals.	Measured in milliseconds (ms)	Continuous
	Percentage of NN intervals greater than 50 ms (pNN50)	Percentage of successive NN (or RR) intervals that differ by more than 50 milliseconds, often used as an indicator of parasympathetic activity.	Measured in percentage (%)	Continuous
	Low frequency (ms^2)	Reflects HR variations within a low frequency span. Supposed to reflect the sympathetic activity of the ANS.	Measured in milliseconds (ms^2)	Continuous
	High frequency (ms^2)	Reflects HR variations within a high frequency span. Supposed to reflect the parasympathetic activity of the ANS.	Measured in milliseconds (ms^2)	Continuous
	LF/HF ratio	Reflects the controlled and balanced behavior of the two branches of the ANS.	Normalized units (n.u.) or absolute power	Continuous
**Psychosocial variables**	Pain catastrophizing	Tendency to magnify the threat value of a pain stimulus and feel helpless in the presence of pain. Measured by the Pain Catastrophizing Scale, a 13-items self-reported survey.	0 to 52 points, higher scores indicate more catastrophic thinking.	Continuous
	Anxiety	How the subject has been feeling in the last week, expressed as common symptoms of anxiety. Measured by the Beck Anxiety Inventory (BAI), a 21-items self-reported survey.	0 to 63 points, higher scores more anxiety.	Continuous

	Depression	Assessment of the presence of several symptoms related to depression. Measured by the Beck Depression Inventory (BDI), a 21-item, self-report survey.	0 to 63 points, higher scores more depressive symptoms.	Continuous
	Cognition	Measured by the Montreal Cognitive Assessment (MoCA) a test that assesses cognitive health by assigning points to various tasks and sections.	0 to 30 points, higher scores indicate less cognitive impairment.	Continuous
	Sleep quality	An individual's overall adequacy of nocturnal rest, encompassing subjective satisfaction, latency, duration, continuity, depth, and restoration, during a typical month. Measured by the Pittsburgh Sleep Quality Index (PSQI) global score, a 7-item, self-reported survey.	0 to 21 points, lower scores indicate better sleep quality, and higher scores indicate worst sleep quality.	Continuous

Figure S1. Distribution of psychosocial variables across PLP–PLS index groups.

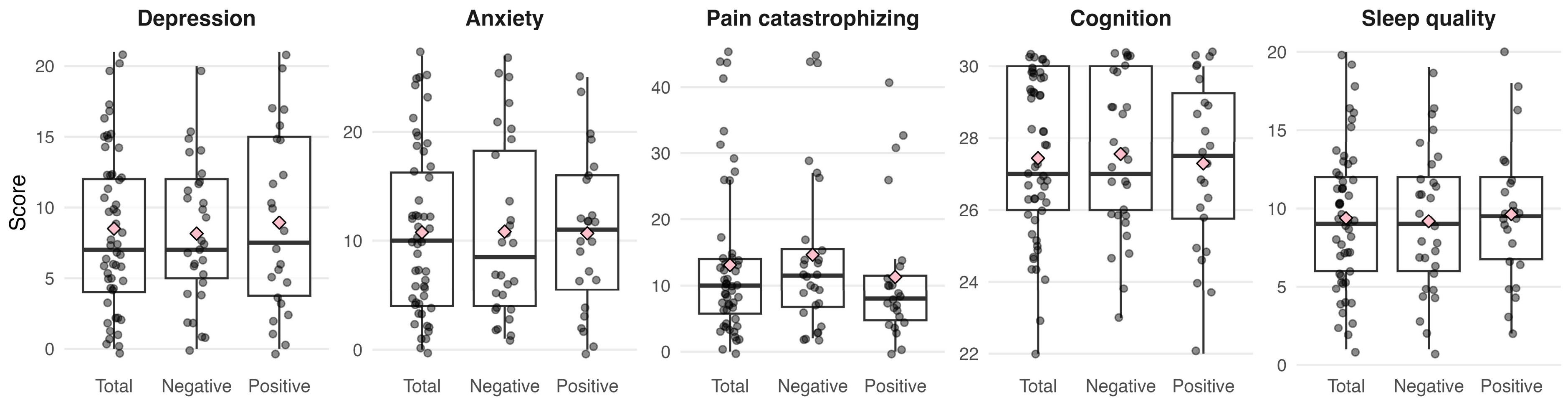

Boxplots show individual and total participant scores for depression, anxiety, pain catastrophizing, cognition, and sleep quality.

Table S2. Univariate analyses for HRV time domain outcomes.

Predictor	Mean RRi		SDNN		RMSSD		pNN50	
	β coefficient	*p-value*	*β coefficient*	*p-value*	*β coefficient*	*p-value*	*β coefficient*	*p-value*
**Age**	2.739	**0.019**	-0.161	0.327	0.056	0.680	-0.017	0.868
**Sex, female**	-40.409	0.278	3.418	**0.217**	2.920	0.405	0.569	0.857
**BMI**	-0.100	0.971	0.796	0.469	1.214	0.510	0.428	**0.063**
**Race**								
White	-71.361	**0.185**	-16.370	0.279	-13.564	0.869	-6.632	**0.159**
African American	-33.123	0.562	-4.230	**0.007**	-3.638	**0.010**	-1.027	0.836
More than 1	-213.789	**0.109**	-20.613	0.845	-19.821	0.966	-6.919	0.547
Asian	-178.789	**0.178**	-10.313	**0.193**	-16.521	**0.253**	-6.919	0.547
**Ethnicity**, Hispanic or Latino	13.587	0.802	-5.419	0.532	-3.856	0.638	-0.709	0.877
**Education level**								
Undergraduate degree	-11.800	0.777	-13.041	0.873	-17.944	0.923	-7.655	**0.029**
Graduate degree	96.536	**0.199**	0.107	0.540	-12.636	0.605	-8.075	**0.192**
**Time since amputation (yrs)**	1.374	0.314	-0.004	0.976	-0.134	0.973	-0.103	0.373
**Left amputation**	-9.672	0.793	8.630	0.993	8.100	0.786	3.733	**0.227**
**Upper amputation**	-1.238	0.977	-1.300	0.984	-3.079	0.914	-1.206	0.744
**Amputation level**								
Partial foot	26.500	0.851	-14.900	0.666	-11.733	0.759	-3.048	0.808
Below the knee	9.079	0.882	8.447	0.267	9.119	**0.166**	3.831	0.483
Knee disarticulation	66.000	0.538	9.550	0.988	11.367	0.803	7.017	0.462
Above the knee (transfemoral)	88.875	**0.161**	4.894	**0.251**	4.479	0.340	2.730	0.625
Hip disarticulation	8.833	0.924	0.867	0.737	-4.700	0.529	-2.872	0.727
Hemipelvectomy/ hindquarter amputation	-138.500	0.330	3.900	0.889	-5.333	0.720	-3.048	0.808
Upper arm/shoulder	-33.000	0.758	-8.450	0.366	-8.783	**0.040**	-3.048	0.749
Lower arm	8.500	0.952	8.000	0.423	3.767	**0.147**	-1.368	0.913
Other	207.500	**0.057**	25.150	0.822	23.767	0.985	15.197	**0.115**
**Amputation reason**								
sepsis	-21.104	0.626	-6.021	**0.090**	3.419	**0.126**	0.133	0.971
oncologic	66.762	**0.252**	-7.852	0.327	-7.362	0.680	-5.550	0.265
vascular, non-diabetic	-97.810	0.322	-17.010	**0.217**	-7.855	0.405	-6.736	0.423
vascular	73.190	0.458	-21.510	0.469	-15.905	0.510	-7.411	0.379
other	-49.310	0.497	-9.260	0.279	-9.280	0.869	-6.909	0.267
**Traumatic amputation**	1.365	0.971	-6.920	**0.007**	-1.157	**0.010**	-2.409	0.442
**Use of pain medication**	-17.291	0.692	-19.803	0.845	-20.749	0.966	-9.147	**0.011**
**Use of prosthesis**	1.932	0.963	12.002	0.327	11.915	0.680	5.825	**0.093**
**PLP frequency**								

Predictor	Mean RRi		SDNN		RMSSD		pNN50	
	β coefficient	*p-value*	*β coefficient*	*p-value*	*β coefficient*	*p-value*	*β coefficient*	*p-value*
A few times a month	-96.000	0.516	5.067	**0.193**	1.200	**0.253**	0.000	1.000
A few times a week	82.818	0.535	30.755	0.532	29.700	0.638	13.021	**0.255**
A few times a day	12.793	0.922	14.262	0.873	11.841	0.923	3.906	0.724
A few times per hour	83.333	0.573	4.133	0.540	2.767	0.605	0.000	1.000
Always	-64.167	0.642	14.850	0.976	13.817	0.973	7.298	0.535
**Phantom limb pain**	0.702	0.334	-0.004	0.993	-0.005	0.786	0.001	0.990
**Phantom sensation**	0.616	0.271	-0.001	0.984	-0.029	0.914	-0.024	0.615
**Residual limb pain**	0.698	**0.238**	-0.002	0.666	0.012	0.759	0.002	0.971
**Telescopic pain**	0.104	0.915	0.072	0.267	0.056	**0.166**	0.052	0.524
**Positive PLP-PLS index**	-40.891	0.263	7.019	0.988	9.559	0.803	5.271	**0.085**
**PLP-PLS index, continuous**	-0.207	0.718	-0.001	**0.251**	0.027	0.340	0.025	0.602
**Pain before amputation, yes**	25.737	0.483	7.259	0.737	6.623	0.529	1.860	0.549
**Psychosocial variables**								
Depression	-2.138	0.511	-0.190	0.889	-0.388	0.720	-0.155	0.574
Anxiety	-1.209	0.617	0.059	0.366	0.166	**0.040**	0.068	0.743
Cognition	-9.725	**0.246**	-1.317	0.423	-3.214	**0.147**	-1.305	**0.063**
Pain catastrophizing	0.708	0.653	0.221	0.822	0.434	0.985	0.162	**0.225**
Sleep quality	-5.581	**0.172**	0.160	**0.090**	-0.015	**0.126**	0.047	0.892

Table S3. Univariate analyses for HRV frequency domain outcomes.

**Predictor**	**Low Frequency**		**High Frequency**		**LF/HF Ratio**	
	β coefficient	*p-value*	*β coefficient*	*p-value*	*β coefficient*	*p-value*
**Age**	-9.476	0.286	-0.232	0.961	-0.115	**0.065**
**Sex, female**	328.199	**0.236**	135.606	0.352	-2.511	**0.200**
**BMI**	16.841	0.415	27.470	**0.009**	-0.058	0.693
**Race**						
White	-541.767	**0.192**	-185.733	0.398	-3.740	**0.186**
African American	-289.386	0.511	-59.114	0.800	0.333	0.911
More than 1	-590.053	0.561	-231.447	0.668	12.924	**0.066**
Asian	-371.053	0.714	-210.447	0.696	5.384	0.437
**Ethnicity**, hispanic or latino	-248.084	0.537	-51.059	0.809	-0.123	0.966
**Education level**						
Undergraduate degree	-363.986	**0.247**	-324.614	**0.047**	2.875	**0.195**
Graduate degree	-60.000	0.915	-296.643	0.303	4.244	0.285
**Time since amputation (yrs)**	-1.871	0.854	-3.030	0.571	0.034	0.639
**Left amputation**	369.591	**0.173**	163.438	**0.253**	3.009	**0.116**
**Upper amputation**	-73.386	0.822	-70.197	0.681	3.868	**0.089**
**Amputation level**						
Partial foot	-286.500	0.802	-74.333	0.901	-7.909	0.314
Below the knee	281.921	0.570	219.140	0.401	-3.812	0.263
Knee disarticulation	63.000	0.942	21.167	0.963	-5.372	0.365
Above the knee (transfemoral)	261.812	0.605	83.729	0.753	-5.247	**0.134**
Hip disarticulation	93.167	0.901	3.333	0.993	-4.296	0.402
Hemipelvectomy/ hindquarter amputation	-24.500	0.983	-53.333	0.929	1.781	0.819
Upper arm/shoulder	-207.500	0.810	-60.833	0.893	2.046	0.729
Lower arm	76.500	0.947	135.667	0.821	-7.849	0.317
Other	970.000	0.264	394.167	0.386	-6.913	**0.245**
**Amputation reason**						
sepsis	-310.647	0.347	127.076	0.466	-5.818	**0.010**
oncologic	-346.714	0.432	-109.286	0.639	-1.231	0.671
vascular, non-diabetic	-708.000	0.345	-138.571	0.726	-7.487	**0.132**
vascular	-684.500	0.361	-170.071	0.667	-6.352	**0.199**
other	-509.000	0.357	-110.821	0.703	0.783	0.829
**Traumatic amputation**	-347.820	**0.205**	24.519	0.866	-3.721	**0.053**
**Use of pain medication**	-906.144	**0.004**	-389.244	**0.019**	2.037	0.376
**Use of prosthesis**	434.916	**0.155**	193.306	**0.230**	-2.793	**0.199**
**PLP frequency**						
A few times a month	82.333	0.940	11.000	0.985	1.196	0.886

Predictor	Low Frequency		High Frequency		LF/HF Ratio	
	β coefficient	*p-value*	*β coefficient*	*p-value*	*β coefficient*	*p-value*
A few times a week	1,218.455	**0.220**	505.727	0.347	0.103	0.989
A few times a day	327.655	0.733	117.621	0.822	2.433	0.741
A few times per hour	39.667	0.971	24.000	0.968	-0.049	0.995
Always	234.333	0.818	120.000	0.828	1.743	0.824
**Phantom limb pain**	-2.226	0.682	-0.364	0.899	0.049	**0.199**
**Phantom sensation**	-2.435	0.560	-1.388	0.527	-0.008	0.783
**Residual limb pain**	-0.036	0.994	-0.024	0.992	0.033	0.286
**Telescopic pain**	0.151	0.983	-0.192	0.960	-0.001	0.979
**Positive PLP-PLS index**	183.254	0.502	210.303	**0.139**	0.436	0.822
**PLP-PLS index, continuous**	1.154	0.787	1.214	0.587	0.039	**0.197**
**Pain before amputation, yes**	320.931	**0.238**	118.739	0.407	-0.110	0.955
**Psychosocial variables**						
Depression	-18.602	0.442	-10.863	0.393	0.228	**0.181**
Anxiety	-11.008	0.546	4.179	0.662	-0.086	0.501
Cognition	-16.312	0.795	-59.680	**0.066**	0.294	0.508
Pain catastrophizing	2.789	0.815	9.894	**0.109**	-0.031	0.711
Sleep quality	10.405	0.734	0.584	0.971	0.509	**0.016**
